# 923. EDP-938, A Respiratory Syncytial Virus (RSV) antiviral, demonstrates a high barrier to resistance in a human challenge study

**DOI:** 10.1093/ofid/ofad500.968

**Published:** 2023-11-27

**Authors:** Rachel E Levene, John DeVincenzo, Annie Conery, Alaa Ahmad, Bryan Goodwin, Yat Sun Or, Michael Rhodin

**Affiliations:** Enanta Pharmaceuticals, Watertown, Massachusetts; Enanta Pharmaceuticals, Watertown, Massachusetts; Enanta Pharmaceuticals, Watertown, Massachusetts; Enanta Pharmaceuticals, Watertown, Massachusetts; Enanta Pharmaceuticals, Watertown, Massachusetts; Enanta Pharmaceuticals, Inc., Watertown, MA; Enanta Pharmaceuticals, Inc., Watertown, MA

## Abstract

**Background:**

RSV can cause substantial morbidity and mortality to populations at high risk and there are no approved direct acting antiviral treatments. EDP-938, an RSV nucleoprotein (N) inhibitor in Phase 2 clinical development, has demonstrated significant reductions in viral load and symptoms after 5 days of dosing in a human challenge study. In contrast to other mechanisms in development, such as fusion inhibitors, EDP-938 has a high barrier to resistance *in vitro*. Here, we characterize the clinical resistance profile from the EDP-938 challenge study.

**Methods:**

To assess emerging resistance, the N gene was sequenced from nasal washes of RSV infected subjects at multiple timepoints before, during and after dosing. A subset of the 124 RSV infected subjects (37 EDP-938 and 11 placebo subjects, 159 total nasal wash samples) were chosen for sequence analysis based on viral load curves to encompass the range of patterns observed over the course of the study. Sequences were compared to baseline (RSV-A Memphis 37) and low frequency variant detection (< 1%) was performed.

**Results:**

Of the 158 sequences obtained, no mutations identified in preclinical *in vitro* resistance studies were observed. Single N-protein mutations were detected in 2 subjects, both of whom received EDP-938. One subject had an E112G mutation detected on day 3 of treatment, however only wildtype virus was subsequently detected, and viral load became undetectable shortly thereafter. The other subject had an L139I mutation detected at days 4 and 5 of treatment and viral load was undetectable the next day. E112G and L139I viral variants were created by reverse genetics and tested for resistance and viral fitness *in vitro*. The E112G mutation did not confer resistance to EDP-938 nor any changes in viral fitness. The L139I mutation conferred very low-level resistance (10-fold decrease in EDP-938 antiviral activity) and a modest reduction in fitness.

**Conclusion:**

In conclusion, no significant clinical resistance to EDP-938 was observed in this Phase 2 challenge study. These data demonstrate that EDP-938 has a high barrier to the development of clinical resistance.

**Disclosures:**

**Rachel E. Levene, PhD**, Enanta Pharmaceuticals: Salary|Enanta Pharmaceuticals: Stocks/Bonds **John DeVincenzo, MD**, Enanta Pharmaceuticals: employee of Enanta Pharmaceuticals **Annie Conery, Ph.D.**, Enanta Pharmaceuticals, Inc.: Stocks/Bonds **Alaa Ahmad, PhD**, Enanta Pharmaceuticals Inc.: Employee|Enanta Pharmaceuticals Inc.: Stocks/Bonds **Bryan Goodwin, Ph.D.**, Enanta Pharmaceuticals: Former Employee|Enanta Pharmaceuticals: Stocks/Bonds **Yat Sun Or, Ph. D**, Enanta Pharmaceuticals: Co-inventor of EDP-938 patents|Enanta Pharmaceuticals: Stocks/Bonds **Michael Rhodin, Ph. D**, Enanta Pharmaceuticals Inc.: Employee of Enanta|Enanta Pharmaceuticals Inc.: Stocks/Bonds

